# Rhizospheric soil microbial community structure and metabolic characteristics of wild *Cymbidium mastersii* at different altitudes

**DOI:** 10.3389/fmicb.2026.1720137

**Published:** 2026-04-01

**Authors:** Mingyu Dang, Ying Tang, Jinxun Chen, Wenwen Xie, Yunfei Zhong, Bo Yu, Erhao Zhang, Zhongbin Wang

**Affiliations:** 1 College of Forestry and Grassland, Xizang Agriculture and Animal Husbandry University, Linzhi, China; 2 The Provincial and Ministerial Co-founded Collaborative Innovation Center for R&D in Xizang Characteristic Agricultural and Animal Husbandry Resources, College of Food Science, Xizang Agriculture and Animal Husbandry University, Linzhi, China; 3 College of Resources and Environment, Xizang Agriculture and Animal Husbandry University, Linzhi, China

**Keywords:** altitude, correlation analysis, *Cymbidium mastersii*, metabolome, metagenome, rhizosphere soil

## Abstract

**Introduction:**

*Cymbidium mastersii*, a perennial orchid of high ornamental value, faces severe survival challenges due to extremely low natural seed germination rates (<15%), habitat degradation, and illegal harvesting. It is listed as a Category II Nationally Protected Plant Species in China.

**Methods:**

We examined the rhizosphere microbial communities and metabolomes of *C. mastersii* across elevation gradients. We investigated the rhizospheric soil microbial community composition and metabolic characteristics of C. *mastersii* across different elevations.

**Results:**

The dominant bacterial phylum was Pseudomonadota, with relative abundances of 38.22% (CmL, low elevation), 36.91% (CmM, mid-elevation), and 62.54% (CmH, high elevation). While the dominant bacterial genera varied significantly with elevation, taxonomic richness exhibited a consistent decline with increasing altitude (*p* < 0.05, linear regression), indicating altitudinal filtering of microbial diversity. LC–MS/MS metabolomic profiling identified 1,516 metabolites, predominantly enriched in lipid and lipid-like molecules, carbohydrates and derivatives, and aromatic compounds. Functional contribution analysis revealed *Bradyrhizobium* as the most influential taxon (10% variance explained), displaying a nonlinear elevational response. Correlation analysis of differential metabolites confirmed significant species-metabolite correlations (*P* < 0.05, *R* > 0.7). Our findings underscore the critical role of trophic interactions in shaping rhizosphere community assembly in alpine plants, thereby contributing to the broader understanding of microbial biogeography along elevational gradients.

**Discussion:**

This study not only confirms that the altitudinal gradient serves as a key environmental filter shaping the rhizosphere microecology of *C. mastersii*, but more importantly, by integrating metagenomic and metabolomic approaches, we systematically reveal for the first time that altitude differentially selects for microbial taxa with specific functions, ultimately driving the restructuring of the rhizosphere metabolic environment. Moving beyond mere community description, our work aims to elucidate the underlying pathways responsible for these shifts and their potential functional implications for host plant adaptation.

## 1 Introduction

*Cymbidium mastersii*, an epiphytic orchid (Orchidaceae) growing on trees or rocks in forest ecosystems (Orchidaceae, 2004, 2009), is classified as a National Grade II Protected Plant in China (Li et al., 2021; Tang et al., 2012) with its primary distribution in southwestern regions. This species holds significant ornamental, economic, and cultural value; however, its wild populations are experiencing severe decline due to intrinsic reproductive constraints (Ohno and Kako, 1978). Current propagation depends predominantly on tissue culture and division techniques (Guo et al., 2012; Liu et al., 2011). Notably, interactions within the rhizospheric microbiome remain an underexplored research frontier essential to holistic conservation strategies.

The rhizosphere is a highly interconnected microzone where plant roots, soil, and microorganisms interact dynamically (Zhang et al., 2019). Soil microorganisms may not regulate isolated metabolic pathways, but rather influence the coordinated regulatory network within plants, leading to synchronous changes in a series of functionally related compounds. The composition and activity of the rhizosphere microbiota are shaped by plant root exudates and, in turn, can modulate the biosynthesis and accumulation of secondary metabolites in the host plant (Oliveira et al., 2014). Microorganisms in the rhizospheric soil, comprising bacterial, fungal, and actinomycetal communities, are regulated by root exudates and establish symbiotic, antagonistic, or neutral relationships with plants, profoundly affecting nutrient acquisition, disease resistance, and growth (Philippot et al., 2013; Dearnaley et al., 2012). Their key functions include facilitating nutrient cycling (Richardson and Simpson, 2011) secreting phytohormones (Berendsen et al., 2012) enhancing stress resilience (Pieterse et al., 2014) mediating symbiotic associations (e.g., orchid seed germination dependent on fungi) (Fay and Krauss, 2003), and degrading pollutants (Araujo, 2022). The composition of rhizosphere microbiota is influenced not only by deterministic factors such as temperature (Zhou et al., 2016), precipitation (Fierer et al., 2003), ultraviolet radiation (Silva et al., 2022), and mycorrhizal symbiosis (Bryant et al., 2008; Xu et al., 2014; Xie et al., 2025; Tedersoo et al., 2020; Van Der Heijden et al., 2008), but also by stochastic processes. However, studies on *C. mastersii*, an orchid species characterized by its unique mycorrhizal symbiosis, are still in their infancy, underscoring the necessity of this research.

Metagenomics has emerged as a powerful approach for advancing research on plant microbe interactions. Microbial metagenomic sequencing involves high-depth sequencing of entire microbial community genomes, offering advantages such as high throughput, rapid processing, and comprehensive genetic information (Alma'abadi et al., 2015). This approach enables accurate characterization of microbial species composition, abundance, functional potential, and metabolic networks, thereby opening new avenues for microbial research (Franzosa et al., 2014). Metagenomic technology is widely used in orchid research. For example, Rühlemann et al. (2023) revealed distinct symbiotic microbial communities through comparative analysis, showing that epiphytic orchids primarily harbor wood-decaying fungi adapted to bark substrates, while terrestrial orchids are predominantly associated with humus-degrading actinobacteria. Similarly, Wang et al. (2024) demonstrated in a study on *Salvia miltiorrhiza* that differences in microbial community structure directly influence plant growth and metabolism, further highlighting the critical role of microbial functions in plant–soil interactions. Metabolomics, in contrast, qualitatively and quantitatively analyzes dynamic changes in metabolites before and after stress exposure, thereby revealing the mechanisms underlying plant disease resistance. An integrated analysis of early interactions between seeds of the orchid *Serapias vomeracea* and its symbiotic fungus *Tulasnella* sp. by Rose et al. (2024) revealed a rapid and specific upregulation of metabolites, including lipids and sterols, in seeds upon fungal contact. This finding elucidates, at the metabolic level, the mechanism by which orchid seeds prepare and remodel their nutritional and signaling environment to facilitate the establishment of symbiosis. Untargeted metabolomics, in particular, compares relative metabolite abundances across samples to identify differential responses (Ros et al., 2020). Metabolomics plays a crucial role in elucidating the mechanisms of plant-environment interactions. Untargeted metabolomics technology, by comparing the relative abundance of metabolites, can accurately identify differential responses of plants to varying environmental conditions (Allwood et al., 2006). This technique not only enables the quantitative analysis of soil metabolites—primarily derived from root exudates and microbial activities—but also correlates with the physicochemical properties of the soil (Ding et al., 2020).

It is important to note that significant knowledge gaps remain in understanding the symbiotic mechanisms between orchids and mycorrhizal fungi, particularly regarding metabolic exchanges (Favre-Godal et al., 2020). Therefore, integrating metabolomics with other multi-omics approaches to systematically analyze root exudates and fungal metabolites will help uncover the chemical dialog underlying these symbiotic relationships.

Current research on *C. mastersii* has primarily focused on tissue culture and rapid propagation, whereas investigations into its rhizospheric soil microbial community composition and metabolite characteristics remain scarce. In this study, we employed metagenomic sequencing and untargeted metabolomics to investigate the effects of different altitudes on the rhizosphere microbial community structure and metabolite profiles of *C. mastersii*, thereby identifying key microbe metabolite interactions. By analyzing the rhizosphere soil microorganisms of *C. masterii*, relevant analytical models were constructed to predict the community composition and functions. Our integrated analysis revealed systematic intraspecific variation in both microbial and metabolic components across the elevational gradient. By constructing relevant models, we identified key microbe-metabolite interactions that were significantly associated with altitude, thereby clarifying how environmental factors orchestrate community assembly and metabolic function.

## 2 Materials and methods

### 2.1 Description of the sampling site

The soil of snow orchids in this study was collected from Dexing Village (CmL), Miri Village (CmM), and Deergong Village (CmH) in Dexing Township, Motuo, Xizang. The longitude, latitude, elevation, and the annual average temperature and precipitation of each sampling site are presented in (Table 1).

**Table 1:** Detailed information on rhizosphere soil samples of *C. mastersii.*

Sample	Longitude	Latitude	Elevation	Mean annual temperature	Annual precipitation
CmL	95.31	29.33	784.34 m	17.5°C	2,500 mm
CmM	95.38	29.41	953.57 m	16.5°C	2,350 mm
CmH	95.15	29.19	1555.57 m	14.5°C	2257.7 mm

### 2.2 Sampling site and soil sample collection

The sampling method followed the protocol described by Tan et al. (2017). In the natural habitat of *C. mastersii*, 10 healthy plants were randomly selected, maintaining a minimum spacing of 100 cm between individuals. A sterile shovel was used to excavate at a distance of 10–15 cm from the plant stem, carefully removing the entire *C. mastersii* plant along with the intact soil block surrounding its root system. The gentle shaking method was applied: gloves were used to lightly tap or shake the root system, allowing large, loose soil not tightly adhered to the roots to fall away naturally. Subsequently, a sterile brush was employed to collect the soil still tightly attached to the fine roots into a sterile sealed bag. All samples were stored in a 4 °C vehicle refrigerator, and processing was completed within 24 h. From each sampling site, three soil replicates were collected, thoroughly homogenized, and divided into triplicate aliquots labeled as CmL1 CmL3 (low altitude), CmM1 CmM3 (mid-altitude), and CmH1 CmH3 (high altitude), then preserved at −80 °C for subsequent analysis.

### 2.3 DNA extraction and metagenomic sequencing

High-quality total genomic DNA was extracted from 500 mg of soil samples using the Soil Genomic DNA Extraction Kit (DP336, Tiangen Biotech, Beijing, China) according to the manufacturer’s protocol (Liu et al., 2023). The integrity of the extracted DNA was verified by 1% agarose gel electrophoresis. Paired-end (PE) libraries were subsequently constructed by ligating Y-shaped adapters, followed by magnetic bead-based purification to remove adapter self-ligation fragments. Library template enrichment was performed via PCR amplification, and single-stranded DNA fragments were generated through sodium hydroxide denaturation. The concentration and purity of the final libraries were assessed using a UV–Vis spectrophotometer before submission to Majorbio Bio-Pharm Technology Co., Ltd. (Shanghai, China) for sequencing. Sequencing was performed on the Illumina NovaSeq 6000 platform (Illumina, San Diego, CA, United States) in paired-end (2 × 150 bp) mode, aiming for a minimum of 10 Gb of raw data per sample. The raw metagenomic sequencing data have been deposited in the NCBI database^1^ under the accession number PRJNA1281867.

For soil samples, we employed an 80% methanol–water solution-based extraction method for metabolite extraction. Briefly, 100 mg of freeze-dried and ground rhizosphere soil sample was accurately weighed, and 100 mg of liquid nitrogen-ground tissue sample was added. The mixture was processed through vortexing, centrifugation, supernatant collection, dilution, and other steps to prepare the test solution. All samples were uniformly analyzed using LC–MS (Dunn et al., 2011; Want et al., 2010). Chromatographic separation was performed using a Hypersil Gold C18 column maintained at 40 °C with a flow rate of 0.2 mL/min. Metabolites were separated via a gradient elution program, with mobile phase A consisting of water containing 0.1% formic acid for positive mode and 5 mM ammonium acetate at pH 9.0 for negative mode, while mobile phase B was methanol in both cases. For quality control, QC samples (prepared by pooling equal volumes from each experimental sample) and blank samples (using 53% methanol–water solution as a substitute for experimental samples, processed identically to the experimental samples) were included (Want et al., 2013).

### 2.4 Statistical analysis of metagenomic data

Raw sequencing data were processed using MEGAHIT (v1.2.9) for *de novo* assembly, retaining contigs >300 bp. Gene prediction was performed with Prodigal (v2.6.3), and functional annotation was obtained by aligning predicted protein sequences against the KEGG database using DIAMOND (v2.1.6, blastp, *e*-value < 1e-5). Taxonomic profiles were generated using MetaPhlAn. All gene and taxon abundances were normalized to relative abundance within each sample prior to downstream analyses. To compare microbial community structure among the three sampling sites (CmL, CmM, and CmH sites), we calculated alpha diversity indices (Sobs, Chao1, Shannon, Simpson) using Mothur (v1.30.2). Differences in alpha diversity among sites were assessed using one-way ANOVA (for normally distributed data) or the Kruskal-Wallis test. Beta diversity was evaluated based on Bray-Curtis and UniFrac distances and visualized via principal coordinate analysis (PCoA) using QIIME (v1.9.1; permutations: *R* = 1, *p* = 0.001). To identify specific taxa with significant abundance differences between sites, linear discriminant analysis effect size (LEfSe) was performed (*p* < 0.05, LDA threshold > 4).

### 2.5 Untargeted metabolomic analysis

The identified metabolites were annotated using the KEGG, HMDB, and LIPIDMAPS databases. Multivariate statistical analyses, including principal component analysis (PCA) and orthogonal partial least squares-discriminant analysis (OPLS-DA), were performed using the metaX software (Wen et al., 2017). Normality of the distribution for each metabolite’s inter-group data was assessed using the Shapiro–Wilk test, and homogeneity of variances was evaluated with Levene’s test. For univariate analysis, the statistical significance (*p*-value) of each metabolite between groups was determined using Student’s *t*-test, alongside the fold change (FC), which represents the relative difference in metabolite abundance. Metabolic pathway analysis was conducted based on the KEGG database, with a pathway considered enriched when x/n > y/n and significantly enriched when *p* < 0.05. Differential metabolites were identified using default thresholds of variable importance in projection (VIP) > 1, *p* < 0.05, and FC ≥ 2 or FC ≤ 0.5. Volcano plots were generated using the ggplot2 package in R, integrating three key parameters VIP values, log2 (FC), and −log10(*p*-value) to identify biologically relevant metabolites. Spearman correlation analysis was conducted to examine relationships between differential metabolites and microbial taxa, the results were visualized using Gephi (0.10.1). Statistical differences were assessed one way analysis of variance (ANOVA) and Duncan’s multiple comparison tests in SPSS 27.0. Significant correlations were defined as those with a *p* < 0.05 and an absolute correlation coefficient (R) > 0.7.

## 3 Results

### 3.1 Effects of altitude on microbial diversity in rhizosphere soil

**Figure 1:**
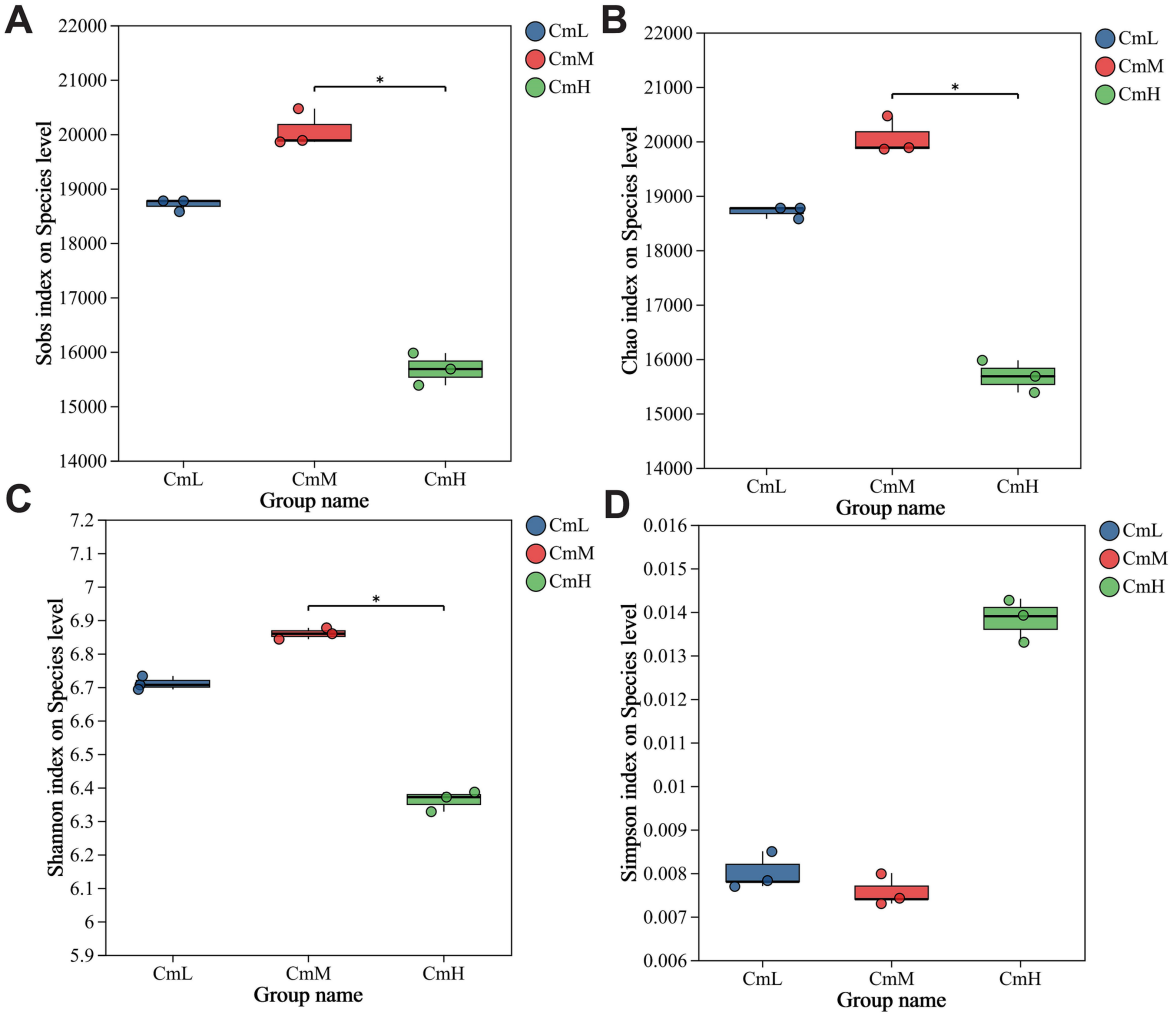
Alpha diversity of soil microorganisms in the rhizosphere of *Cymbidium mastersii*. The analysis employed richness indices [Sobs index **(A)** and Chao index **(B)**] and diversity indices [Shannon index **(C)** and Simpson index **(D)**], CmL stands for low altitude, CmM stands for medium altitude, and CmH stands for high altitude, with significance levels set at *p* < 0.05.

Sequencing results showed that a total of 416,599,842 raw reads were obtained from nine samples. After quality control, 411,271,954 clean reads remained, with detailed information for each sampling site presented in Table 2. The assembled sequences comprised 61.964 Gb of bases, forming 2,309,414 contigs with an average length of 424.16 bp. Richness analysis showed that the CmM site exhibited the highest microbial richness, followed by CmL, while CmH displayed the lowest values, with CmM being significantly richer than CmH (Figures 1A,B). Diversity index data indicated that CmM maintained the highest microbial diversity, whereas CmH showed the lowest (Figures 1C,D). Significant differences in rhizosphere microbial alpha diversity were detected in the rhizosphere soil of *C. mastersii*. These findings demonstrate that altitude gradients significantly influence α diversity patterns in the rhizosphere microbial communities of *C. mastersii*.

**Table 2:** Metagenomic raw data.

Sample ID	Raw reads (#)	Raw base (GB)	Clean reads (#)	Clean base (GB)	Percent in raw reads (%)	Contigs	ORFs^a^	Average length of ORFs (bp)
CmL1	45,683,772	6.898	45,110,410	6.796	98.74493288	232,542	260,322	378.05
CmL2	47,611,748	7.189	47,005,066	7.082	98.72577247	226,840	255,841	379.36
CmL3	47,216,064	7.129	46,589,662	7.019	98.67332864	215,017	243,718	381.76
CmM1	49,356,182	7.452	48,709,926	7.338	98.69062806	260,979	297,309	388.6
CmM2	45,544,324	6.877	44,910,212	6.766	98.60770356	209,046	238,766	390.65
CmM3	44,974,588	6.791	44,312,408	6.676	98.52765744	206,816	235,664	390.07
CmH1	48,882,668	7.381	48,321,872	7.281	98.85277129	250,071	279,959	387.03
CmH2	44,673,260	6.746	44,166,032	6.655	98.86458253	236,437	264,252	385.01
CmH3	42,657,236	6.441	42,146,366	6.351	98.80238373	209,548	233,583	386.51

a The starting gene fragment that may encode proteins in DNA sequences.

**Figure 2:**
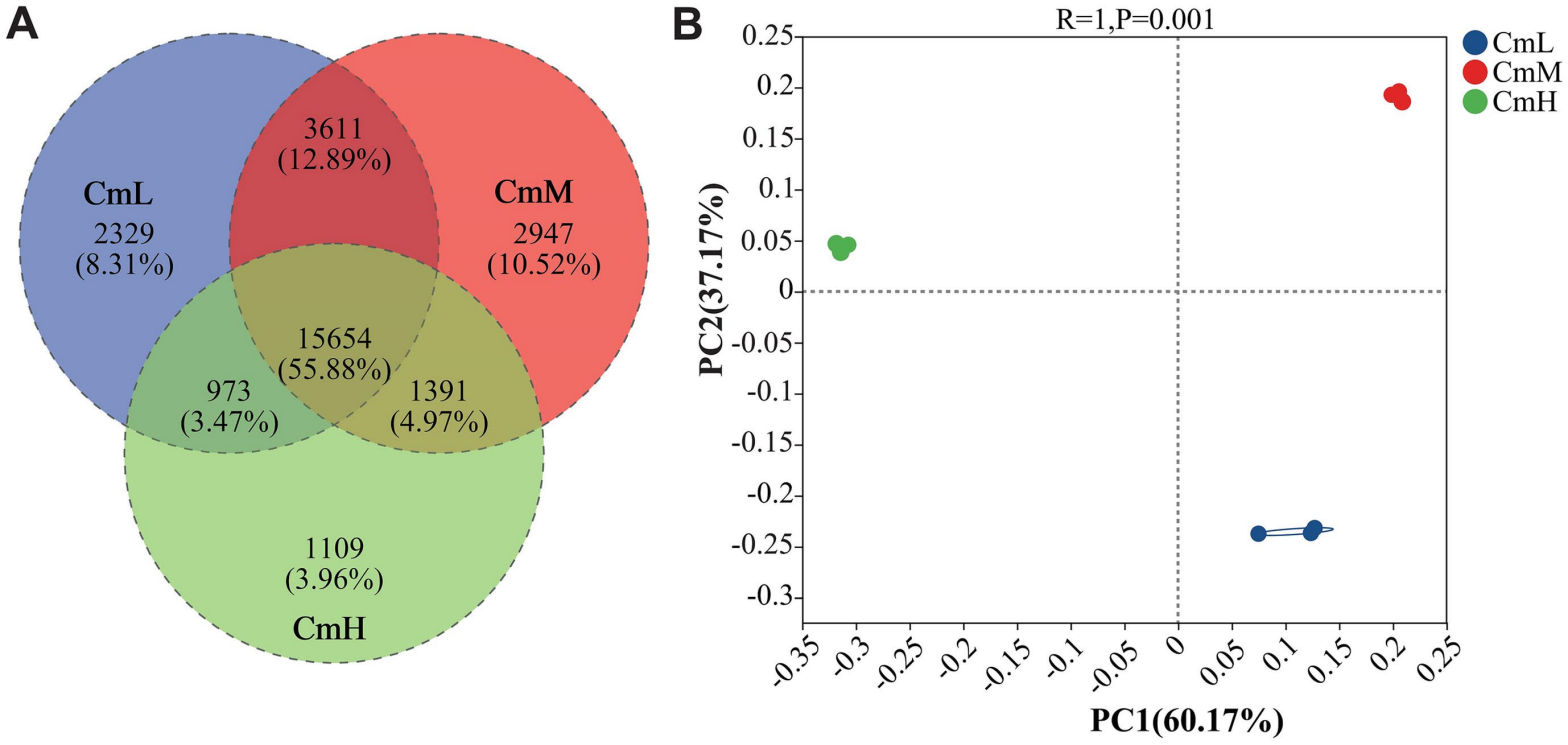
Species composition and β-diversity patterns across different altitudinal sites. **(A)** Venn diagram illustrating the proportions of shared and unique species among CmL, CmM, and CmH sites. **(B)** Principal coordinates analysis (PCoA) plot showing significant intergroup variation (*p* < 0.01). CmL stands for low altitude, CmM stands for medium altitude, and CmH stands for high altitude.

Venn diagrams and PCoA were employed to investigate the effects of altitude on microbial community composition. As shown in Figure 2A, a total of 22,567, 23,603, and 19,127 were detected species in the CmL, CmM, and CmH sites, respectively. Of these, 15,654 species (55.88%) were shared across all sites, while site-specific species accounted for 2,329 (8.31%) in CmL, 2,947 (10.52%) in CmM, and 1,109 (3.96%) in CmH. The Venn diagram analysis demonstrated distinct rhizosphere microbial community compositions of *C. mastersii* at different altitudes. The PC1 and PC2 explained 60.17 and 37.17% of microbial community variation, respectively, (Figure 2B). The three sampling sites were located in distinct quadrants with substantial spatial separation, indicating significant compositional differences in rhizosphere microbial communities. Within altitudes, these results confirmed that altitudinal gradients substantially influence the structural organization of microbial communities in *C. mastersii* rhizosphere soils.

### 3.2 Effects of altitude on microbial community composition in rhizosphere soil

**Figure 3:**
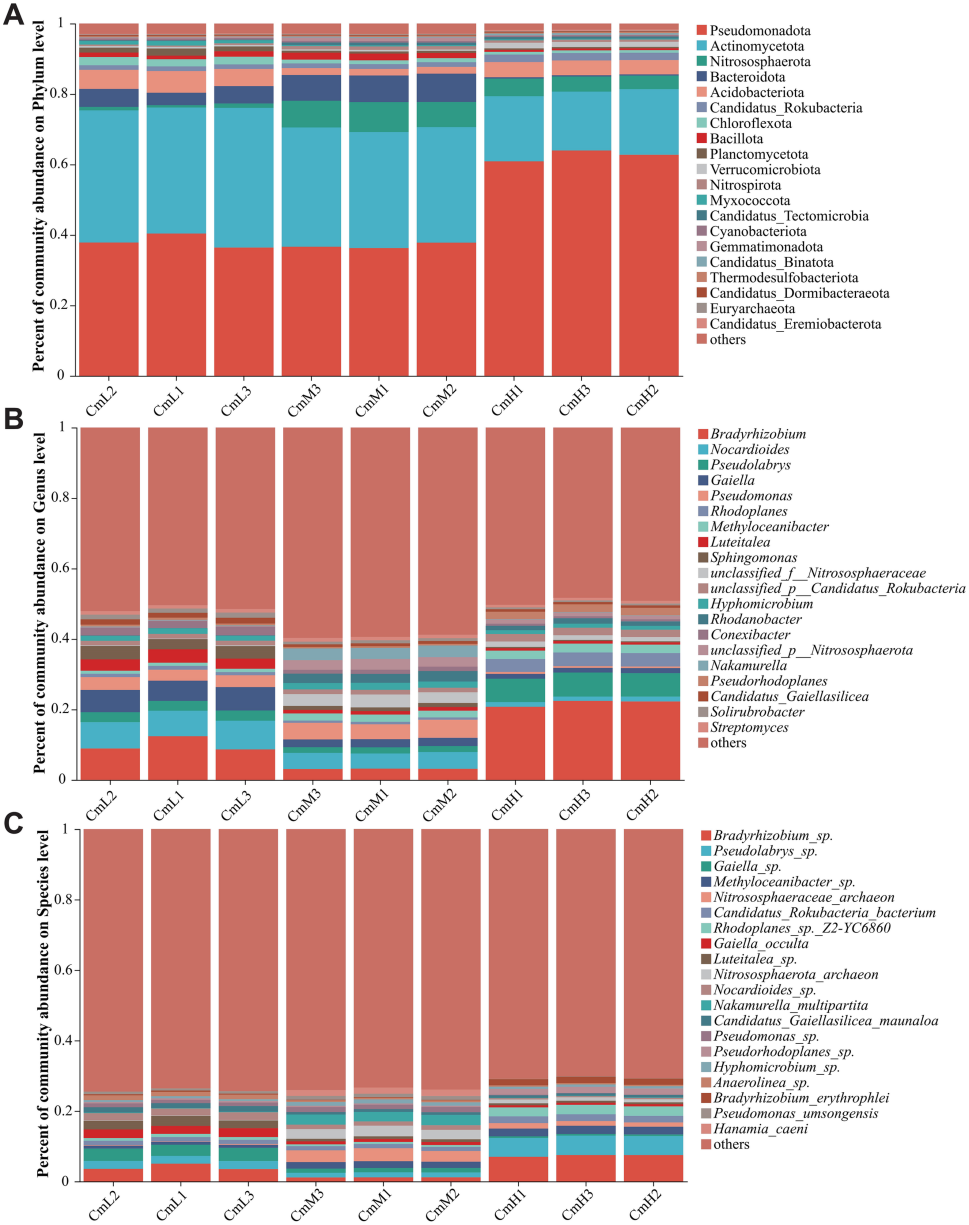
Taxonomic composition of microorganisms in the rhizosphere of *Cymbidium mastersii*, based on NR database annotations, showing the relative abundance profiles at the **(A)** phylum, **(B)** genus, and **(C)** species levels, with only the top 20 most abundant taxa displayed at each taxonomic rank. In the figure, a larger color proportion for each column indicates a higher relative abundance. CmL stands for low altitude, CmM stands for medium altitude, and CmH stands for high altitude.

Taxonomic annotation against the NR database identified a total of 222 phyla, 381 classes, 661 orders, 1,263 families, 4,389 genera, and 28,014 species across all samples. At the phylum level (relative abundance >1%) (Figure 3A), the CmL site was dominated by nine phyla, including Pseudomonadota (38.22%), Actinomycetota (37.64%), and Acidobacteriota (5.47%). The CmM site was dominated by eight phyla, including Pseudomonadota (36.91%), Actinomycetota (33.19%), and Nitrososphaerota (7.74%). The CmH site was dominated by six phyla, including Pseudomonadota (62.54%), Actinomycetota (17.92%), and Nitrososphaerota (4.38%). Notably, Pseudomonadota was the most abundant phylum across all sites, while Actinomycetota consistently ranked second but displayed a clear decline in relative abundance with increasing elevation, from 37.64% (CmL) to 17.92% (CmH). This pattern suggests a significant negative correlation between Actinomycetota abundance and altitude. As shown in Figure 3B at the genus level (relative abundance >1%), microbial communities across sampling sites exhibited distinct taxonomic profiles. The dominant genera in the CmL plot mainly consisted of 15 genera, including *Bradyrhizobium* (10%), *Nocardioides* (7.6%), and *Gaiella* (6.23%). The dominant genera in the CmM plot mainly consisted of 13 genera, including *Pseudomonas* (4.66%), *Nocardioides* (4.54%), and *Nakamurella* (3.26%). In contrast, the dominant genera in the CmH plot mainly consisted of 14 genera, including *Bradyrhizobium* (21.82%), *Pseudolabrys* (6.7%), and *Rhodoplanes* (3.79%). These results reveal significant altitudinal variations in dominant microbial taxa, suggesting that elevation gradients strongly influence the compositional structure of rhizosphere microbial communities in *C. mastersii*. Figure 3C illustrates distinct microbial community compositions at the species level (relative abundance >1%) across different altitude sites. The CmL site and CmM contained 13 dominant species, while the CmH plot contained 11. As detailed in Table 1, the rhizosphere soil of *C. mastersii* at the low altitude CmL site stood out for its greatest microbial community richness. This trend corresponded to the site’s distinct climatic profile, where both mean annual temperature and precipitation reached their highest levels. These results demonstrate significant altitudinal variations in the composition of dominant microbial species, confirming that elevation gradients substantially influence both the community structure and richness of the rhizosphere soil microbiome of *C. mastersii.*

### 3.3 Species analysis and functional differences in different rhizosphere microbiota

**Figure 4:**
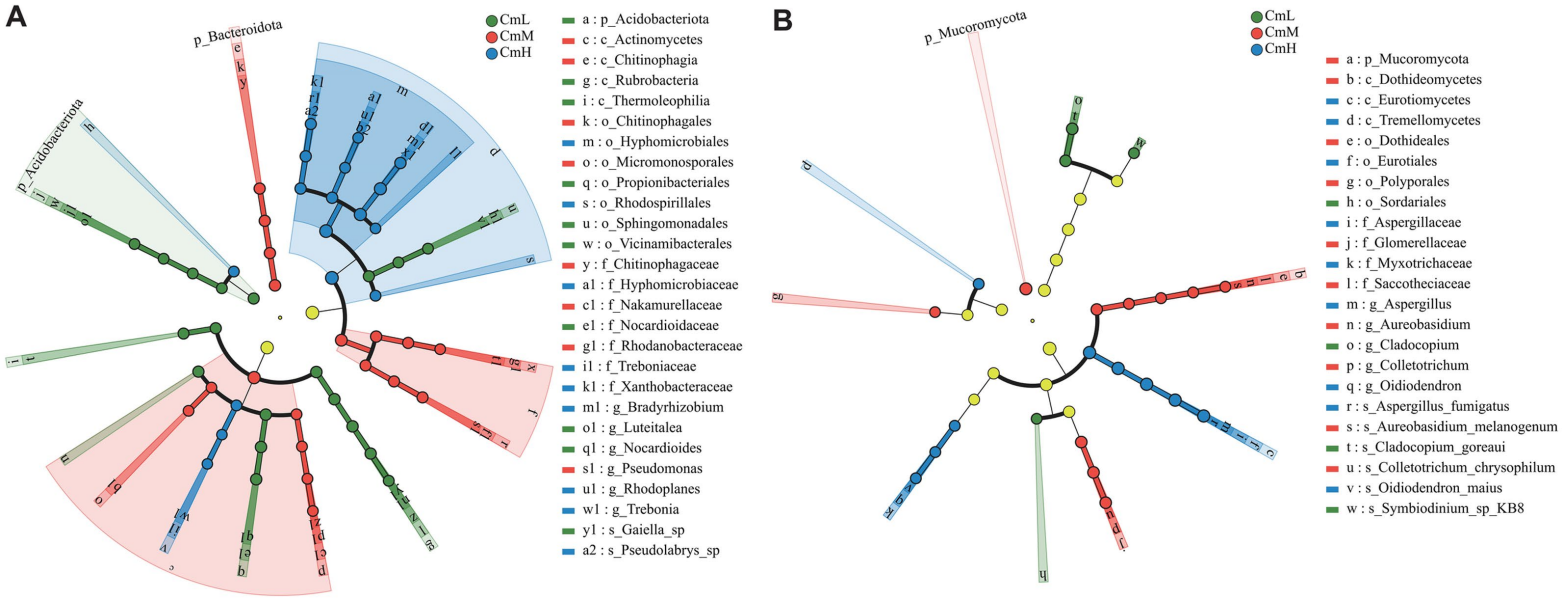
Figure 4 LEfSe results showing differentially abundant bacterial **(A)** and fungal **(B)** taxa (LDA score >4). The phylogenetic tree diagram illustrates taxonomic differences across hierarchical levels, providing a visual representation of differentially abundant taxa identified between groups at various taxonomic ranks. Nodes with distinct colors represent microbial taxa that are significantly enriched in the corresponding groups and play a key role in driving inter-group differences. Specifically, pale yellow nodes indicate non-significant taxa. CmL stands for low altitude, CmM stands for medium altitude, and CmH stands for high altitude.

LEfSe analysis with an LDA threshold >4 identified 54 biomarker taxa, spanning phylum to species levels, in the rhizosphere soil microbiome of *C. mastersii* across different altitudes. The top 20 differential metabolic pathways at the bacterial level are illustrated in Figure 4A. Specifically, at the CmL site, significantly enriched bacterial taxa included Acidobacteriota, Rubrobacteria, *Nocardioides*, and 16 other groups. At the CmM site, significant enrichment was observed for Bacteroidota, Actinomycetes, *Pseudomonas*, along with 15 additional taxa. At the CmH site, significantly enriched taxa comprised Alphaproteobacteria, Terriglobia, *Bradyrhizobium*, and 15 others. A total of 20 biomarker taxa were identified at the bacterial level across the three sampling sites, as shown in Figure 4B. Specifically, the biomarker for the CmL site was Sordariales. For the CmM site, 10 biomarker taxa were identified, while 9 were found at the CmH site. These findings demonstrate significant altitudinal variation in microbial biomarker communities associated with the rhizosphere soils of *C. mastersii*.

**Figure 5:**
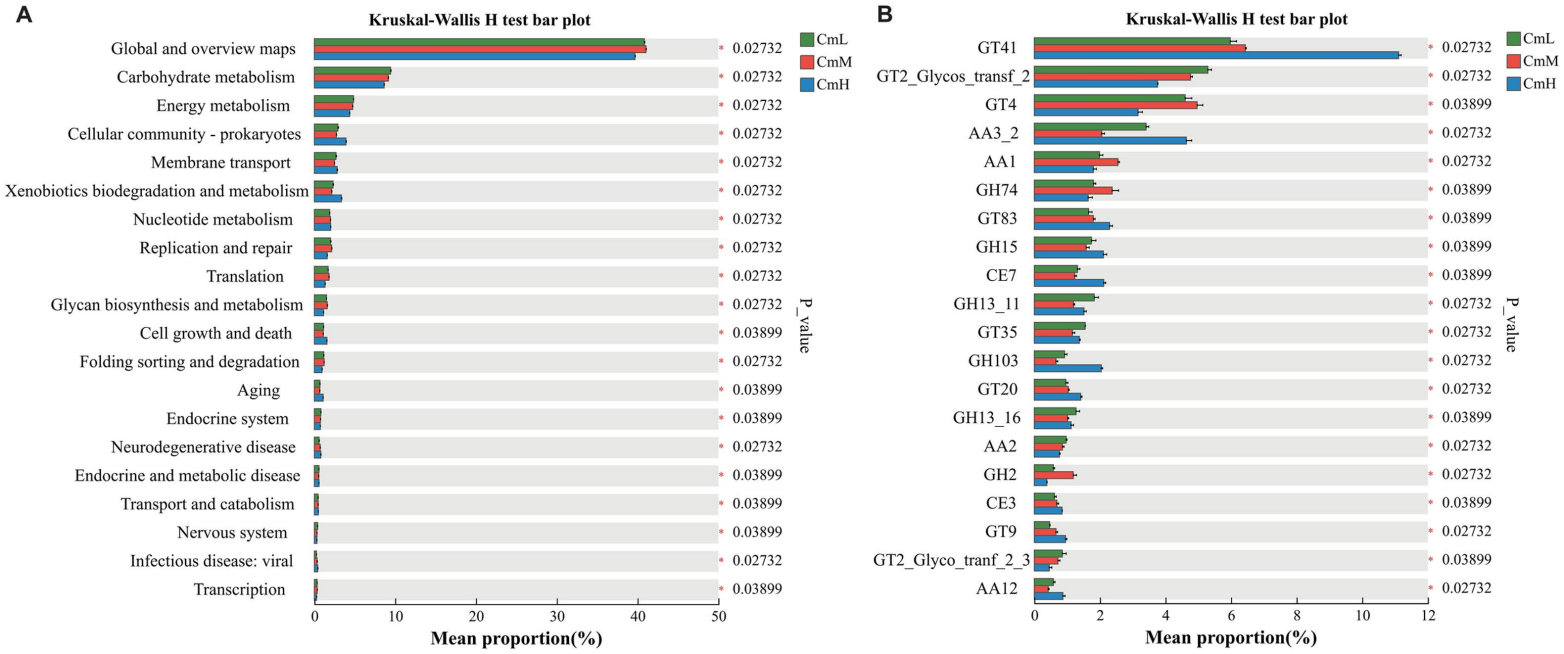
Functional profiling of rhizosphere microbial communities, displaying: **(A)** The top 20 significantly differentiated metabolic pathways at KEGG level 2, and **(B)** carbohydrate-active enzymes (CAZy) classification statistics, highlighting the top 20 enzyme families with significant differences. Panel B emphasizes four major CAZy categories—glycosyltransferases (GT), auxiliary activities (AA), glycoside hydrolases (GH), and carbohydrate esterases (CE)—which play crucial roles in carbohydrate metabolism and modification. CmL stands for low altitude, CmM stands for medium altitude, and CmH stands for high altitude.

To investigate the altitudinal effects on the functional potential of *C. mastersii* rhizosphere microbiota, we analyzed the top 20 significantly differentiated metabolic pathways at KEGG level 2. As shown in Figure 5, the numbers of significantly differential metabolites identified at the CmL, CmM, and CmH sites were 5, 6, and 9, respectively. Collectively, these results indicate that elevation gradients exert profound influences on the functional metabolism of microbial communities in *C. mastersii* rhizosphere soils. At KEGG Level 3, comparative analysis of the top 20 differential metabolic pathways revealed distinct functional profiles across altitudinal gradients in *C. mastersii* rhizosphere microbiomes (Supplementary Figure S1). These findings demonstrate altitudinal stratification of microbial metabolic potential, with lower elevations (CmL) specializing in xenobiotic degradation, mid-elevations (CmM) emphasizing amino acid metabolism, and higher elevations (CmH) prioritizing translational machinery and biosynthesis.

Analysis of species contributions to KEGG metabolic pathways (Supplementary Figure S2) revealed that the relative abundance of the top 10 species in dominant metabolic pathways exhibited a distinct altitudinal pattern: initially decreasing from low to mid elevation, followed by an increase, peaking at high altitude. Bradyrhizobium emerged as the primary contributor, displaying a characteristic U-shaped distribution, with its lowest contribution at mid-altitude (CmM) and highest representation at the high-altitude site (CmH). Pseudolabrys, the secondary contributor, exhibited a similar trend of initial decline followed by an elevation-dependent increase. In contrast, *Gaiella*, *G. occulta*, and *Luteitalea* demonstrated a progressive decrease in metabolic contributions along the elevational gradient from CmL to CmH. These findings highlight significant altitudinal variations in both the taxonomic composition and functional potential of rhizosphere microbial communities associated with *C. mastersii*, reflecting adaptive metabolic specialization across different elevation zones.

### 3.4 Effects of altitude on microbial functional metabolism

**Figure 6:**
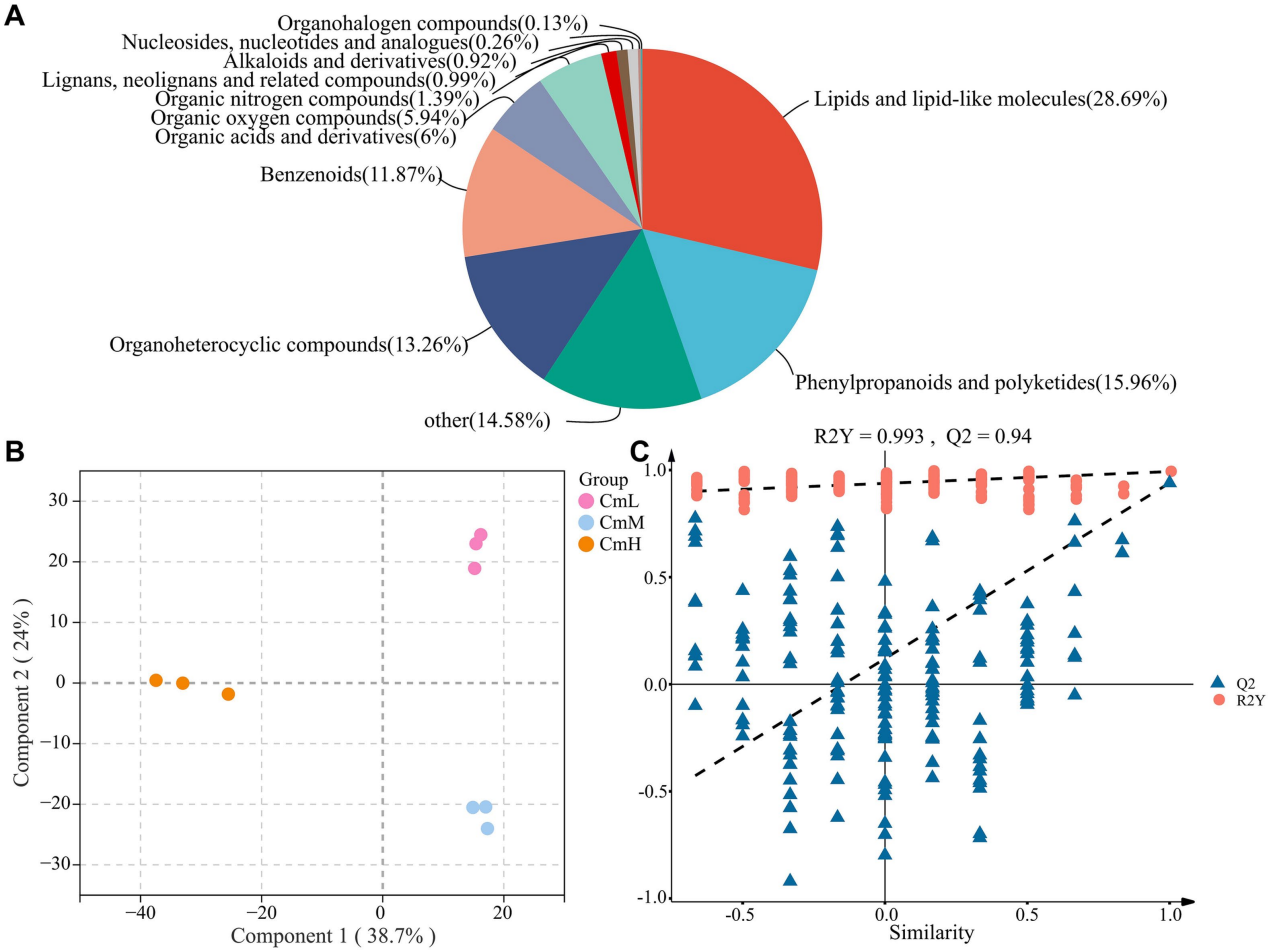
Classification of 12 metabolic compounds **(A)**, principal component analysis **(B)** (Component 1 = 38.7%, Component 2 = 24%), and the OPLS-DA analysis **(C)**, indicating (R^2^Y = 0.7551, Q^2^ = 0.94). R^2^Y represents the degree to which the model explains the variance in the original data (goodness-of-fit), while Q^2^ denotes the predictive capability of the model based on cross-validation (the higher the value, the greater the model’s accuracy in predicting new samples). CmL stands for low altitude, CmM stands for medium altitude, and CmH stands for high altitude.

**Figure 7:**
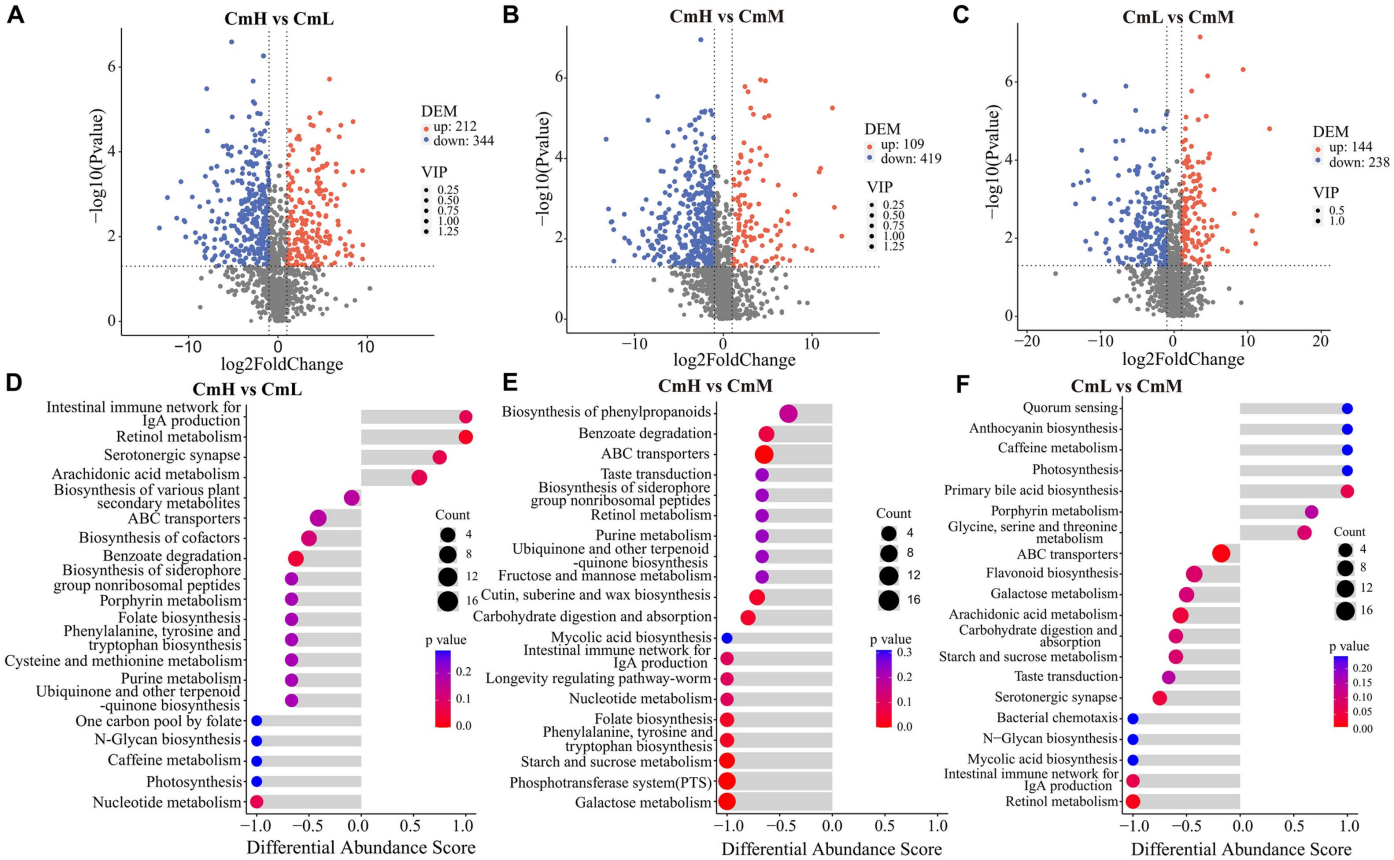
Differential metabolite analysis for the comparisons of CmH versus CmL **(A)**, CmH versus CmM **(B)**, CmL versus CmM **(C)** (VIP values > 1, *p*-values < 0.05, fold changes >2 or <0.5). Up/downregulation indicates higher/lower metabolite content in latter vs former groups.Numbers of differential metabolites are shown. Pathway enrichment analysis **(D-F)**: dot color represents significance (redder=lower *p*-value); dot size indicates metabolite count in each pathway. CmL stands for low altitude, CmM stands for medium altitude, and CmH stands for high altitude.

The untargeted metabolomic analysis of *C. mastersii* rhizosphere soils across different elevations (Supplementary Table S1) identified 1,516 metabolites, comprising 738 in positive ion mode and 778 in negative ion mode. Based on chemical classification (Figure 6A), the metabolites were categorized into 12 classes. The most abundant were lipids and lipid-like molecules (28.69%), phenylpropanoids and polyketides (15.96%), and organoheterocyclic compounds (13.26%). Principal component analysis (PCA) of soil (Figure 6B) revealed significant separation among sampling sites, with a total explained variance of 62.7% (PC1 = 38.7%, PC2 = 24%). OPLS-DA modeling (Figure 6C) demonstrated excellent validity, with R^2^Y = 0.7551 and *Q*^2^ = 0.94. Comparative analysis (VIP > 1, *p* < 0.05, FC > 2 or <0.5) revealed distinct sets of differential metabolites between each pair of sampling sites, as shown in Figures 7A–C. As shown in Figures 7D–F, pathway enrichment analysis (*p* < 0.05) revealed elevation specific patterns. Functional genes associated with ABC transporters were significantly identified and demonstrated high abundance across all three elevation groups (CmL, CmM, CmH). This indicates that transmembrane transport processes, particularly the transport of nutrients, secondary metabolites, or stress-responsive molecules, play a fundamental and crucial role in the ecological functioning of the *C. mastersii* rhizosphere microbiome. These results demonstrate significant elevational gradients in both metabolite diversity and biochemical pathway activation within *C. mastersii* rhizospheres.

**Figure 8:**
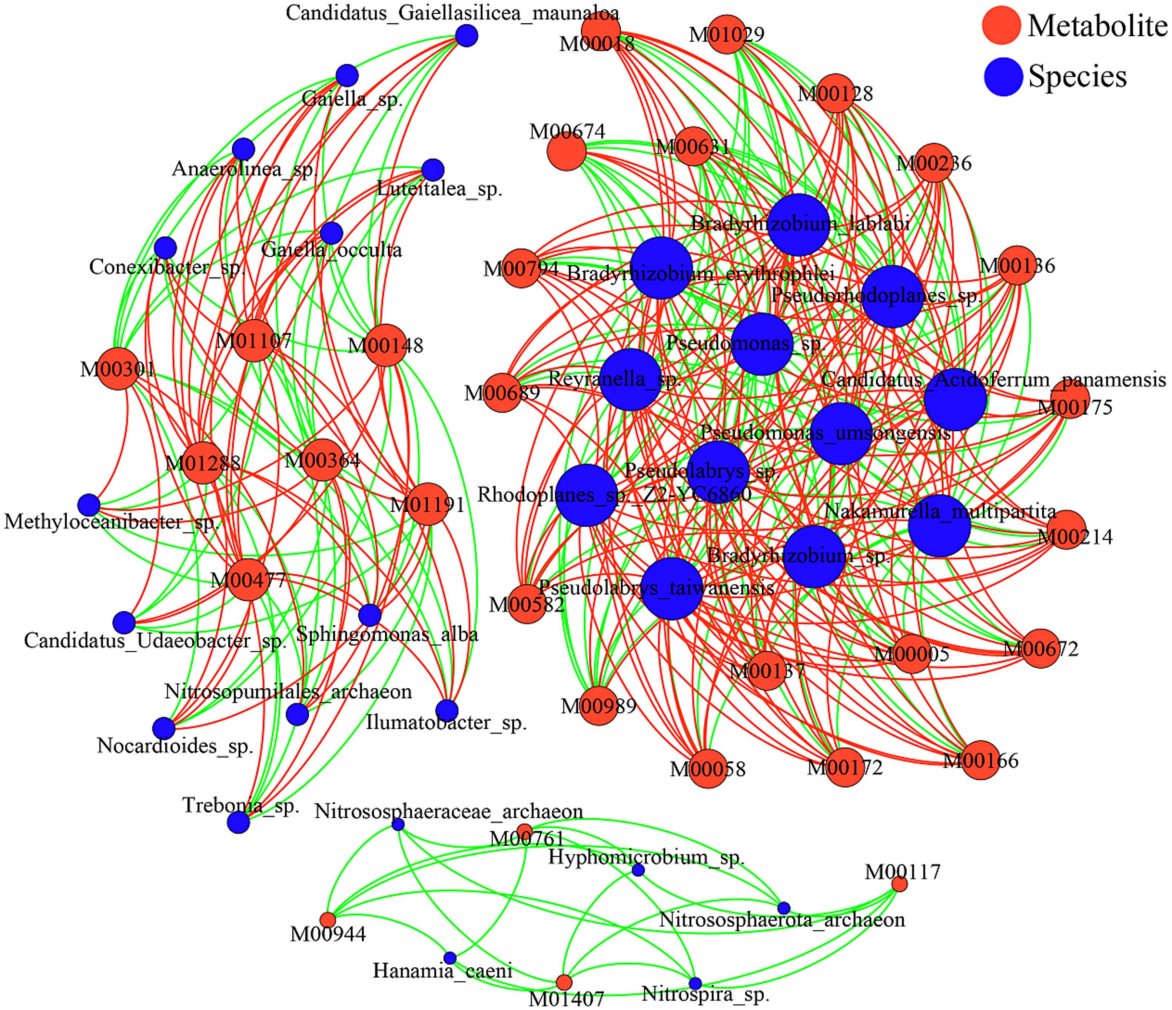
Correlation network between the top 30 species and their metabolites. Red represents positive correlation, and green represents negative correlation. The size of each node reflects its degree of connectivity (the number of edges). Larger nodes indicate a greater number of significant associations involving that particular microbial taxon or metabolite.

To investigate the relationships between rhizosphere soil microorganisms and soil metabolites in *C. mastersii*, we performed a correlation analysis (*p* < 0.05, *R* > 0.7) between the top 30 differential microbial species and the top 30 differential metabolites. As shown in Figure 8, network analysis revealed significant correlations between these microbial species and metabolites, with positive correlations (57.52%) being more prevalent than negative correlations (42.48%). Several notable interactions were observed: *Nakamurella multipartita* exhibited negative correlations with isomaltose, cellobiose, and lactose; *Reyranella* sp. was negatively correlated with 4-fluoro-5-(4-methylpiperazin-1-yl)benzene-1,3-diamine and 3-chloro-4-fluorophenylhydrazine; *Bradyrhizobium erythrophlei* showed positive correlations with isomaltose, cellobiose, lactose, galactinol, 6-demethoxytangeretin, and 9-oxooctadecanoic acid; and *Pseudorhodoplanes* sp. was positively associated with isomaltose, cellobiose, and lactose. These results highlight the complex interrelationships between microbial communities and soil metabolites in the *C. mastersii* rhizosphere across different elevations.

## 4 Discussion

This study systematically unravels the multidimensional scenario of coordinated adaptation to an elevational gradient in *C. mastersii* rhizosphere, integrating metagenomics and untargeted metabolomics to elucidate the interplay among microbial community structure, functional potential, and metabolic environment. In our alpha diversity analysis, the Shannon index peaked at the mid-elevation site, while the Simpson index reached its lowest value at the same location. Therefore, this pattern provides valuable ecological insight. The Shannon index is more sensitive to species richness (the number of species), while the Simpson index is more heavily weighted toward species evenness (the relative abundance of each species) (Bassey et al., 2023). Consequently, our results indicate that the mid-elevation site possesses the highest number of microbial species, a finding further supported by the high values of the Sobs and Chao1 indices. This ‘mid-elevation peak’ in richness is a common pattern in biogeography, often attributed to optimal environmental conditions coupled with reduced competition or environmental stress (Lomolino, 2001). In the context of orchids, this zone may represent an optimal balance between temperature, moisture, and nutrient availability, thereby fostering a highly diverse microbial community. This finding aligns with patterns previously reported for other alpine plants, such as *Rhododendron* (Debnath et al., 2016), *Cymbidium faberi*, and *Cymbidium goeringii* in the Qinling region (Wen et al., 2025). β-diversity analysis was conducted to assess the differences among rhizosphere microbiomes across different elevations. The mid-altitude CmM site containing the highest number of unique species and the high-altitude CmH site the fewest. Venn diagrams were used to visualize the shared and unique species of rhizosphere microorganisms at different elevations. β-diversity analysis revealed significant differences in the composition of rhizosphere soil microbial communities of *C. mastersii* across different elevations, which aligns with findings from previous studies (Bi et al., 2021). This further supports the conclusion that elevation plays a key role in shaping rhizosphere soil microbial community structure. Collectively, these results confirm that altitude significantly influences the abundance, richness, and diversity of *C. mastersii* rhizosphere soil microorganisms, corroborating previous findings in plant–microbe elevation studies.

Through metagenomic analysis of *C. mastersii* rhizosphere soils, we investigated how altitude influences microbial diversity and composition. Harsh physical conditions (e.g., low temperature) and potential habitat fragmentation likely enhance the role of dispersal limitation, preventing many species from the regional species pool from reaching or establishing in these areas, thereby constraining local diversity. In contrast, as an ecotone, this zone offers the most suitable conditions with moderate environmental stress and may benefit from species dispersal from both higher and lower elevation communities. Our results demonstrate significant altitudinal effects on the structure of rhizosphere microbial communities, consistent with the findings of previous studies (Liu et al., 2024). Consistent with multiple studies conducted on the Tibetan Plateau, our results further corroborate the prevalent role of Pseudomonadota as a core phylum and its contrasting responses along elevational gradients, which aligns with the findings reported by Yang et al. (2022). Actinobacteria represents the second most dominant phylum. Previous studies utilizing 16S rRNA sequencing analysis have confirmed that *Actinomycetota* consistently functions as one of the predominant microbial groups in soils across varying elevations and in the rhizosphere microbial communities of subalpine meadows on the Tibetan Plateau (Li et al., 2009; Li et al., 2022). The pronounced enrichment of Pseudomonadota at high elevations may not merely reflect a quantitative abundance shift, but rather result from functional selection. Key functional genera within this phylum include *Pseudomonas* and *Burkholderia*. Their enrichment can be attributed to their metabolic versatility in carbon processing (Chamara et al., 2024), nitrogen cycling capabilities (Knief et al., 2012), and co-evolutionary relationships with plants (Tsavkelova et al., 2007). These findings are consistent with our results. Actinobacteria, the second most abundant phylum, play a major role in carbon cycling and biocontrol within orchid-associated soils (Zhao et al., 2021; Bryant et al., 2008). Key genera include *Streptomyces*, *Micromonospora*, and *Frankia*, with Actinobacteria serving as the dominant group (Li et al., 2023). Tiwari et al. (2022) observed a significant enrichment of Actinobacteria, including the genus Streptomyces, in the rhizosphere. These actinobacteria are likely to assist the host plants in resisting heavy metal stress. This finding directly demonstrates that under contaminated conditions, orchids actively recruit beneficial actinobacteria to their rhizosphere. These microbes presumably aid plant survival through mechanisms such as detoxification and immobilization of heavy metals. We observed a significant negative correlation between the relative abundance of Actinobacteria and elevation. However, similar phenomena reported in previous orchid studies have not provided robust theoretical support for this pattern, whereas this general trend has been confirmed in bulk soil, root microbiomes, or alpine ecosystems. This “one-up, one-down” pattern clearly reflects the reorganization of core functional modules in the rhizosphere microbiome driven by the elevational gradient. It is important to note that phylum-level taxa encompass ecological diversity; however, the consistent shifts in dominant phyla observed here align with broad functional trends reported for the orchid rhizosphere (Xian et al., 2025; Xie et al., 2020). Higher-resolution analyses in the future could further elucidate specific microbial roles.

Understanding how rhizosphere metabolites mediate soil–microbe interactions provide critical insights into the feedback mechanisms that promote plant growth (Xu et al., 2014; Zhang et al., 2022). Principal component analysis (PCA) revealed significant differences in rhizosphere soil metabolites across different elevation gradients. Subsequent orthogonal partial least squares-discriminant analysis (OPLS-DA), based on variable importance in projection (VIP) scores and *p*-values, confirmed its robust predictive capability for screening differential metabolites. This approach led to the identification of a substantial number of significantly altered metabolites. The microbial communities at low elevations (CmL) predominantly exhibited an outward-oriented metabolic profile, characterized by enhanced Lipopolysaccharide biosynthesis, Aromatic compound degradation, and Phosphotransferase system (PTS). This pattern suggests an adaptive strategy centered on intensifying the breakdown of exogenous substrates and membrane transport processes. Sugars are further channeled through primary metabolic pathways into diverse precursor molecules for secondary metabolism. At mid-elevations (CmM), the functional core shifts toward genetic information processing and amino acid metabolism, including translation, replication repair, and the degradation of amino acids such as leucine. This suggests that the mid-elevation zone may represent an ecological niche with relatively abundant energy and stable environmental conditions, where microbial communities invest resources in growth, maintenance, and protein synthesis. At high elevations (CmH), the functional profile is dominated by biosynthetic pathways, ribosome-related processes, and prokaryotic community formation, including amino acid biosynthesis and prokaryotic cellular community pathways. This strongly indicates that under high-elevation stressors such as low temperature and hypoxia, microorganisms enhance protein synthesis capacity and form tightly interacting communities to sustain survival and collective resilience. The dominant contribution of *Bradyrhizobium* is highly consistent with this adaptive strategy. Notably, *Bradyrhizobium* functions not only as a nitrogen fixing bacterium but also as a well characterized plant growth promoting rhizobacterium. Its absolute dominance at high elevations strongly suggests that *Bradyrhizobium* plays a critical role as a symbiotic partner or key probiotic in aiding *C. mastersii* to adapt to extreme environmental conditions. Collectively, these findings align with previous reports (Ramya et al., 2020; De Rose et al., 2023; Walitang et al., 2019). Notably, ABC transporters—which facilitate mycorrhizal symbiosis by mediating carbon and nutrient exchange (e.g., nitrogen, phosphorus) and recognizing fungal secretions (e.g., oligosaccharides, fatty acids)—emerged as key players. These results are consistent with the findings reported by Kovalchuk et al. (2015) and Li et al. (2025). Collectively, these findings demonstrate that rhizosphere metabolites enhance plant growth both directly and indirectly through complex microbe-mediated mechanisms.

Significant correlations exist between microbial communities and differential metabolites (Wang et al., 2019), with microorganisms functioning as key drivers of soil metabolic processes (Li et al., 2020). Our study demonstrates distinct associations between differential metabolites and microbes in the rhizosphere soil of *C. mastersii*. Metabolites showing positive correlations primarily included lipids and their derivatives, carbohydrates and their derivatives, and aromatic compounds. Notably, cellobiose a microbial carbon source plays a pivotal role in driving carbon cycling and influencing soil organic matter decomposition (Bidartondo, 2005; Ilmen et al., 1997). Specifically, cis-9,10-epoxystearic acid acts as a signaling molecule that suppresses toxin production by activating fungal G-protein pathways, thereby enhancing plant defense. Collectively, these findings indicate that extensive interactions between carbohydrates, lipids, and microorganisms promote plant growth, aligning closely with our research outcomes.

## 5 Conclusion

Integrating metagenomic and metabolomic analyses, this study revealed distinct rhizosphere soil microbial communities and metabolite profiles of *C. mastersii* across different elevations. The rhizosphere microbial community structure changed significantly along the elevational gradient, dominated by Pseudomonadota and Actinobacteria. Notably, the relative abundance of Actinobacteria declined with increasing elevation. Correspondingly, the expression of metabolite profiles and associated metabolic processes in the rhizosphere soil also shifted markedly, with lipids and carbohydrates showing the most pronounced changes. Significant correlations were observed between microbial community structure and metabolite composition. These research findings provide the first systematic analysis of the rhizosphere soil microorganisms of *C. mastersii*, establishing a theoretical foundation for its conservation and artificial cultivation, and offering technical support for subsequent industrial production.

## Data Availability

The raw metagenomic sequencing data have been deposited in the NCBI database (https://www.ncbi.nlm.nih.gov/bioproject/) under the accession number PRJNA1281867.

## Footnotes

1
https://www.ncbi.nlm.nih.gov/bioproject/
