# Supplementary material for: Rhizospheric soil microbial community structure and metabolic characteristics of wild Cymbidium mastersii at different altitudes
Source: Front Microbiol. 2026 Apr 1;17:1720137. doi: 10.3389/fmicb.2026.1720137 (PMC13079301; doi:10.3389/fmicb.2026.1720137)
Supplement: Supplementary file 1 [file Data_Sheet_1.doc]

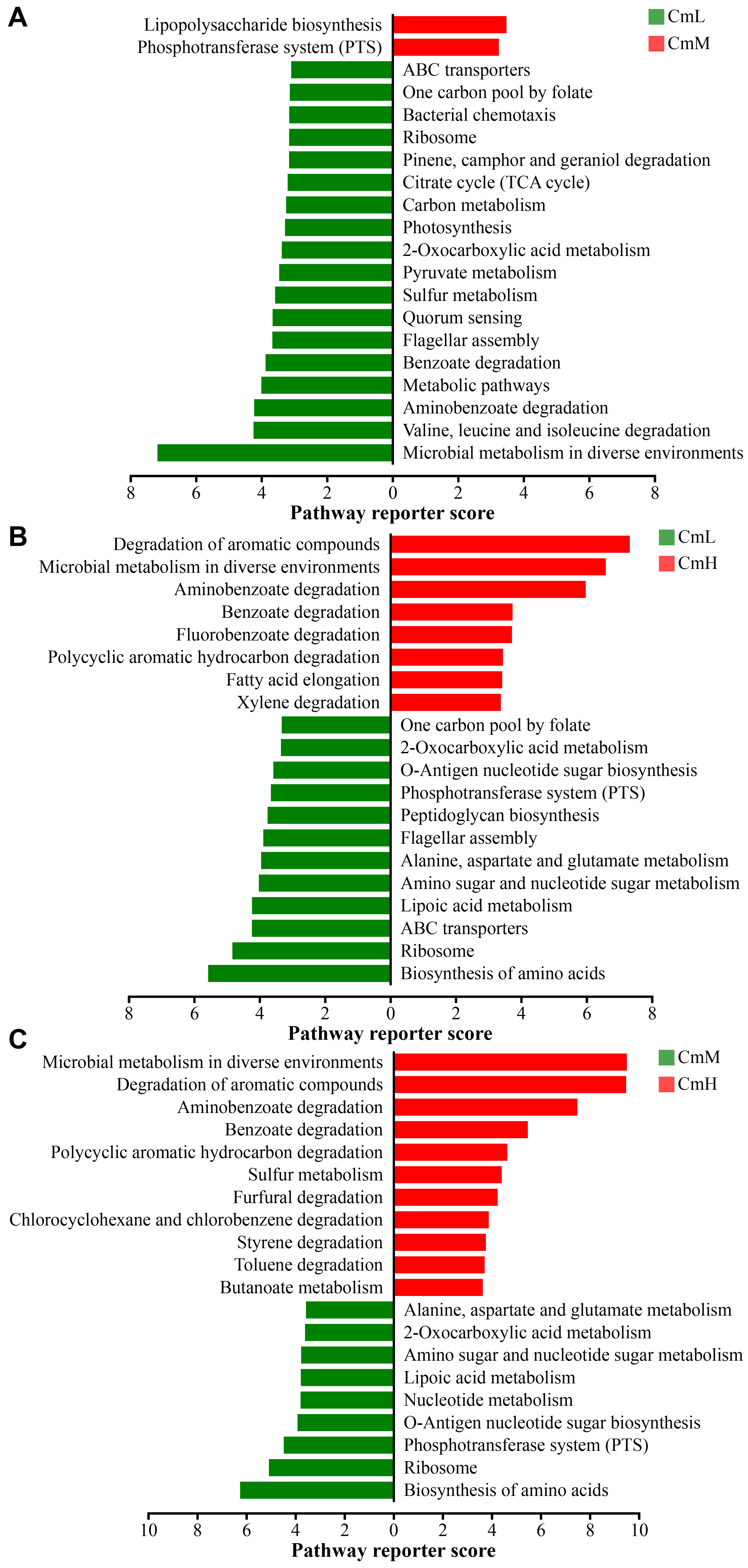


**Figure s1** Comparative analyses of metabolic pathways at KEGG level 3, showing (A) CmL versus CmM, (B) CmL versus CmH, and (C) CmM versus CmH


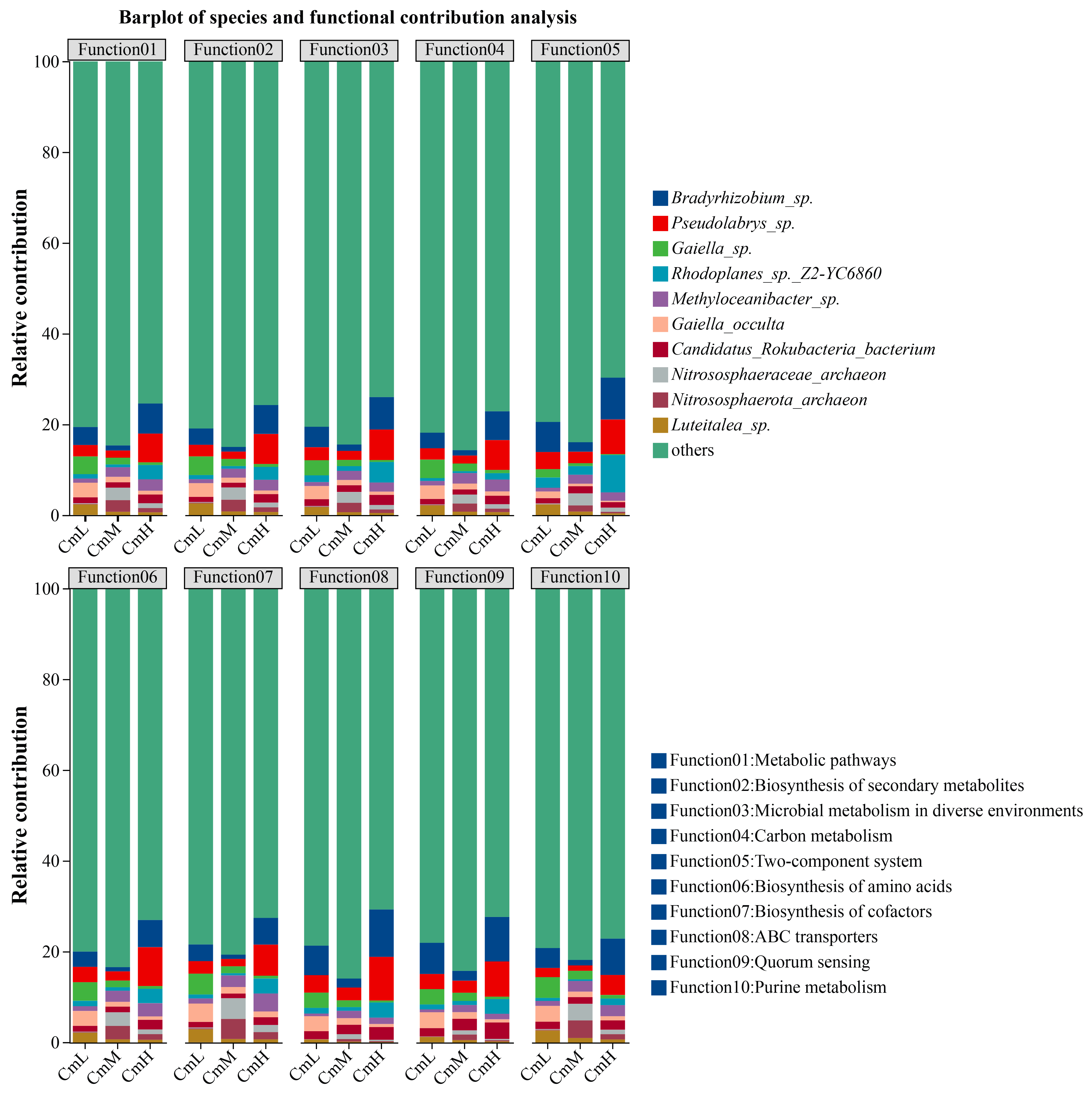


**Figure s1** KEGG contribution analysis of the top 10 species' metabolic pathways

**Table s1** Relative abundance of the top 30 metabolites

| Name | CmL1 | CmL2 | CmL3 | CmM1 | CmM2 | CmM3 | CmH1 | CmH2 | CmH3 |
| --- | --- | --- | --- | --- | --- | --- | --- | --- | --- |
| s__Bradyrhizobium_sp. | 4.042418726 | 3.171102756 | 3.171102756 | 1.145503525 | 1.123798119 | 1.123798119 | 7.318359479 | 7.023853175 | 7.612865784 |
| s__Pseudolabrys_sp. | 2.231639364 | 2.181211625 | 2.181211625 | 1.369947516 | 1.319880878 | 1.319880878 | 5.427547555 | 5.359488149 | 5.427547555 |
| s__Gaiella_sp. | 3.482155094 | 3.209632943 | 3.209632943 | 1.238792919 | 1.195652549 | 1.195652549 | 0.448606558 | 0.442491595 | 0.448606558 |
| s__Methyloceanibacter_sp. | 0.82915721 | 0.813280072 | 0.845034348 | 1.850208816 | 1.79152838 | 1.79152838 | 2.23852804 | 2.154651387 | 2.322404693 |
| s__Nitrososphaeraceae_archaeon | 0.184996068 | 0.132047382 | 0.132047382 | 3.354076981 | 3.01936006 | 3.01936006 | 1.374548545 | 1.191831878 | 1.557265213 |
| s__Rhodoplanes_sp._Z2-YC6860 | 0.81962809 | 0.746030309 | 0.89322587 | 0.535047358 | 0.508709926 | 0.508709926 | 2.652686556 | 2.582852076 | 2.652686556 |
| s__Nitrososphaerota_archaeon | 0.124944097 | 0.105655248 | 0.105655248 | 2.810493942 | 2.614927897 | 2.614927897 | 0.968018042 | 0.866379569 | 1.069656515 |
| s__Gaiella_occulta | 2.406433813 | 2.257101728 | 2.257101728 | 0.850779586 | 0.821073707 | 0.821073707 | 0.589725113 | 0.559413583 | 0.620036643 |
| s__Luteitalea_sp. | 2.497043998 | 2.152824604 | 2.152824604 | 0.752818576 | 0.726128041 | 0.726128041 | 0.557797666 | 0.5357153 | 0.557797666 |
| s__Nocardioides_sp. | 2.071912811 | 1.968562448 | 1.968562448 | 1.270505623 | 1.207876943 | 1.207876943 | 0.391082798 | 0.37409392 | 0.391082798 |
| s__Nakamurella_multipartita | 0.160599106 | 0.159112173 | 0.159112173 | 2.825458036 | 2.714772005 | 2.714772005 | 0.106440832 | 0.096069731 | 0.106440832 |
| s__Candidatus_Gaiellasilicea_maunaloa | 1.489112947 | 1.356658682 | 1.356658682 | 0.760408462 | 0.71151644 | 0.71151644 | 0.679594836 | 0.646928607 | 0.679594836 |
| s__Pseudomonas_sp. | 1.019846833 | 0.938327257 | 0.938327257 | 1.387907265 | 1.255005639 | 1.255005639 | 0.137356953 | 0.117131709 | 0.137356953 |
| s__Pseudorhodoplanes_sp. | 0.404115727 | 0.369280796 | 0.369280796 | 0.353883929 | 0.333499275 | 0.333499275 | 1.704832527 | 1.673382352 | 1.704832527 |
| s__Hyphomicrobium_sp. | 0.564690617 | 0.514807424 | 0.514807424 | 1.073195757 | 0.990636601 | 0.990636601 | 0.651443812 | 0.635718288 | 0.651443812 |
| s__Bradyrhizobium_erythrophlei | 0.315749746 | 0.271873897 | 0.271873897 | 0.149621525 | 0.141804355 | 0.141804355 | 1.788224234 | 1.72598575 | 1.788224234 |
| s__Anaerolinea_sp. | 1.305547702 | 1.217873632 | 1.217873632 | 0.510222144 | 0.490973708 | 0.490973708 | 0.382536045 | 0.3761249 | 0.382536045 |
| s__Pseudomonas_umsongensis | 0.674735292 | 0.618436807 | 0.618436807 | 1.038409356 | 0.9665863 | 0.9665863 | 0.082215525 | 0.070369964 | 0.094061087 |
| s__Hanamia_caeni | 0.01143973 | 0.010422342 | 0.012457117 | 1.755734611 | 1.687007648 | 1.687007648 | 0.016367908 | 0.015855047 | 0.016880769 |
| s__Pseudolabrys_taiwanensis | 0.505729816 | 0.485622028 | 0.485622028 | 0.257149773 | 0.243656305 | 0.243656305 | 1.014547613 | 1.006470499 | 1.014547613 |
| s__Candidatus_Acidoferrum_panamensis | 0.31583116 | 0.284613152 | 0.347049168 | 0.180580541 | 0.173883856 | 0.173883856 | 1.181381457 | 1.175182098 | 1.181381457 |
| s__Nitrosopumilales_archaeon | 0.094928716 | 0.066756603 | 0.123100829 | 0.710766973 | 0.639278142 | 0.639278142 | 0.866771042 | 0.728836891 | 1.004705193 |
| s__Conexibacter_sp. | 0.915572104 | 0.821205411 | 0.821205411 | 0.437281538 | 0.414113926 | 0.414113926 | 0.237267531 | 0.22270389 | 0.237267531 |
| s__Ilumatobacter_sp. | 1.038237725 | 0.941649945 | 0.941649945 | 0.383579833 | 0.356661749 | 0.356661749 | 0.13026616 | 0.123396567 | 0.137135695 |
| s__Sphingomonas_alba | 1.444925222 | 1.267626059 | 1.267626059 | 0.0569646385139 | 0.04839297 | 0.065536307 | 0.015755866 | 0.014192519 | 0.017319213 |
| s__Reyranella_sp. | 0.392488102 | 0.3594746 | 0.3594746 | 0.272525683 | 0.26654461 | 0.26654461 | 0.835315353 | 0.819100452 | 0.835315353 |
| s__Nitrospira_sp. | 0.248096123 | 0.230193533 | 0.230193533 | 0.749066775 | 0.717056058 | 0.717056058 | 0.490731774 | 0.465925533 | 0.490731774 |
| s__Trebonia_sp. | 0.101998796 | 0.094169056 | 0.094169056 | 0.154919664 | 0.154441918 | 0.154441918 | 1.20596056 | 1.128695001 | 1.283226119 |
| s__Candidatus_Udaeobacter_sp. | 0.121950952 | 0.11491341 | 0.11491341 | 0.208817119 | 0.198507469 | 0.219126769 | 0.979647298 | 0.926272266 | 0.979647298 |
| s__Bradyrhizobium_lablabi | 0.355903441 | 0.289301211 | 0.289301211 | 0.107710444 | 0.105742278 | 0.105742278 | 0.805112031 | 0.757645484 | 0.805112031 |
